# Application of loop-mediated isothermal amplification combined with lateral flow assay visualization of *Plasmodium falciparum* kelch 13 C580Y mutation for artemisinin resistance detection in clinical samples

**DOI:** 10.1016/j.actatropica.2023.106998

**Published:** 2023-10

**Authors:** Wannida Sanmoung, Nongyao Sawangjaroen, Suwannee Jitueakul, Hansuk Buncherd, Aung Win Tun, Supinya Thanapongpichat, Mallika Imwong

**Affiliations:** a Division of Biological Science, Faculty of Science, Prince of Songkla University, Hat Yai, Songkhla 90110, Thailand; b Haematology Unit, Department of Medical Technology and Pathology, Suratthani Hospital, Surat Thani Province, Thailand; c Faculty of Medical Technology, Prince of Songkla University, Hat Yai, Songkhla 90110, Thailand; d Faculty of Graduate Studies, Mahidol University, Salaya, Nakhon Pathom 73170, Thailand; e Department of Molecular Tropical Medicine and Genetics, Faculty of Tropical Medicine, Mahidol University, 10400, Thailand; f Mahidol-Oxford Tropical Medicine Research Unit, Faculty of Tropical Medicine, Mahidol University, Bangkok, Thailand

**Keywords:** Plasmodium falciparum, C580Y mutation, LAMP-SNP assay, Lateral flow assay

## Abstract

Resistance to the antimalarial drug artemisinin (ART) has emerged in Greater Mekong Subregion. The molecular marker predominantly used to identify ART resistance is the C580Y mutation in *Pfkelch13* of *Plasmodium falciparum*. Rapid and accurate detection of ART resistance in the field is necessary to guide malaria containment and elimination interventions. Our study evaluates the *Pf*C580Y by using the loop-mediated isothermal amplification and single nucleotide polymorphism analysis visualization using a lateral flow assay (LAMP-SNP-LFA) method for detecting ART resistance in clinical samples collected from Thailand between 2014 and 2019. The optimized incubation condition for the reaction was determined as 45 min at 56 °C, followed by visual detection of positive amplicons using LFA. The assay demonstrated high analytical sensitivity and specificity, with a limit of detection of 16.8 copies of C580Y plasmid/µL of and 100% accuracy for C580Y mutation detection. The *Pf*C580Y LAMP-SNP-LFA method is faster and simpler than conventional polymerase chain reaction/DNA sequencing and has the potential to support antimalarial management policies, malaria control, and global elimination efforts.

## Highlights

• *Pf*C580Y LAMP-SNP-LFA method was developed to detect *Pf*kelch13 mutations.
• This rapid and accurate tool effectively detects *Pf*kelch13 mutations in clinical samples.
• It reduces dye-induced non-specific binding and amplification inhibition in the LAMP assay.
• It can aid in monitoring the spread of drug-resistant infections.

## 1 Introduction

Artemisinin (ART)-based combination therapies are the preferred treatment for *Plasmodium falciparum* malaria. ART is particularly effective against the parasite when combined with other antimalarial drugs, such as lumefantrine, amodiaquine, mefloquine, sulphadoxine–pyrimethamine, piperaquine, pyronaridine, and napthoquine, and has played a significant role in the successful treatment of malaria. However, the emergence of drug-resistant parasite strains, particularly those resistant to ART and its derivatives, is a major global health concern (WHO, 2015a). In recent years, there have been reports of ART resistance and delayed clearance of the falciparum parasite from the blood of patients with malaria in Greater Mekong Subregion (GMS). These reports suggest that mutations in *Pfkelch13*, which codes for kelch 13 (K13) protein involved in the endocytosis of host cell cytosol during the ring stage of the parasite, are responsible for this resistance. Ten mutations (F446I, N458Y, M476I, Y493H, R539T, I543T, P553L, R561H, P574L, and C580Y) in the C-terminal propeller domain of K13 have been confirmed as causes of drug resistance. Of them, the C580Y mutation is associated with ART resistance, which is commonly dominant in GMS and poses a growing concern in this region because it threatens the effectiveness of ART-based therapies. The spread of ART resistance in GMS may significantly impact public health, leading to increased rates of malaria-related morbidity and mortality. Therefore, monitoring the prevalence of the C580Y mutation is crucial in the fight against malaria in GMS (Ariey et al., 2014; Birnbaum et al., 2020; Dondorp et al., 2009; Kobasa et al., 2018; Menard et al., 2016; Noedl et al., 2008; Straimer et al., 2015; Tun et al., 2015). To combat this issue, understanding the genetic basis of drug resistance and developing new diagnostic detection strategies, such as rapid diagnostic tests and molecular diagnostic techniques, can help in the early detection of drug-resistant strains of malaria. Several methods are available to detect single nucleotide polymorphisms (SNPs) and evaluate their association with antimalarial resistance. Typically, protocols involve either DNA sequencing or restriction fragment length polymorphism of polymerase chain reaction (PCR) amplicons and real-time PCR (WHO, 2015b; WHO, 2009). In research and reference laboratory settings, nested PCR amplification and subsequent sequence data analysis are widely regarded as the gold standard for detecting SNPs associated with ART resistance (Menard et al., 2016; Takala-Harrison et al., 2015; Witkowski et al., 2010). However, these methods have limitations, including requiring specialized equipment and a relatively long turnaround time, making them unsuitable for point-of-care testing in remote or resource-limited regions.

Loop-mediated isothermal amplification (LAMP) technology has been utilized to detect the presence of various pathogens, such as malaria parasites, fungi, viruses, and bacteria (Areekit et al., 2022; Gomez-Gutierrez and Goodwin, 2022; Lee et al., 2020; Mohon et al., 2016; Ortega et al., 2020; Srisawat and Panbangred, 2015; Tang et al., 2011; Xiao and Li, 2021; Yin et al., 2016). LAMP technology can be used for SNP detection using the mismatch approach. This entails adding the SNP base to the 3ʹ end of a LAMP primer to obstruct polymerase extension in the presence of non-SNP sequences (Badolo et al., 2012b; Duan et al., 2016; Liu et al., 2018). LAMP-based SNP detection has been demonstrated in various studies, highlighting its potential applications for genotyping, drug resistance testing, and clinical diagnosis (Badolo et al., 2012a; Ding et al., 2019; Lin et al., 2018; Yongkiettrakul et al., 2017). Although LAMP technology has been noted for its high specificity and sensitivity for SNP detection, the colorimetric dyes in LAMP detection can have downsides, including, amplification inhibition, nonspecific binding, interference with downstream applications, and increased cost (Silva et al., 2019). Therefore, alternative methods, such as lateral flow assays (LFAs), have been developed to detect LAMP products, eliminating the need for dyes. The LFA test strip comprises capture lines coated with specific antibodies or other molecules. A colored line on the test strip is observed if the labeled LAMP product binds to the capture lines.

The aim of this investigation was to evaluate the performance of the *Pf*C580Y LAMP-SNP-LFA method on clinical samples from patients with malaria to determine its potential as an alternative diagnostic tool for both point-of-care testing and molecular surveillance of markers of antimalarial drug resistance. This method has the potential to offer a portable and feasible solution for detecting antimalarial resistance markers in regions where malaria is endemic.

## 2 Materials and methods

### 2.1 Blood sample collection

This study analyzed 100 genomic DNA samples (*P. falciparum*, 91; *P. vivax*, 8, and *P. malariae*, 1) collected between 2014 and 2019 from patients with malaria. The majority of the samples were collected from three provinces that have international borders with Thailand, namely, the Thai-Cambodian, Thai-Myanmar, and Thai-Malaysian borders. *P. falciparum* genomic DNA was extracted and purified from 62 samples of whole blood collected in Ubon Ratchathani province, which is located near the Thai-Cambodian border (Imwong et al., 2015). The remaining 38 genomic DNA samples were extracted from dried fingerpick blood spots. Of these, 26 samples (*P. malariae*, 1, and *P. falciparum*, 25) were collected from Ranong province, which is close to the Thai–Myanmar border. Additionally, 12 samples (*P. falciparum*, 4, and *P. vivax*, 8) were collected from Yala province in the southern region of Thailand (Khammanee et al., 2019). The extracted DNA was purified using a QIAamp DNA Blood Mini Kit (Qiagen, Hilden, Germany) following the manufacturer's protocol, and the extracted DNA was stored at −20 °C.

### 2.2 Primers used for the *Pf*C580Y LAMP-SNP-LFA method

In this study, we used five primers that recognized seven distinct regions covering the C580Y mutation in *Pfkelch13*. The external primers (F3 and B3) and internal primers (FIP and BIP) were previously described (Khammanee et al., 2021). To enhance the specificity for C580Y mutation detection, we introduced a loop primer (LF) that included a single mismatched nucleotide at the penultimate position of the primer's 3ʹ terminus. Additionally, the 5ʹ ends of FIP and BIP were labeled with fluorescein isothiocyanate (FITC) and biotin, respectively (Table 1).

**Table 1:** Primers used in our study.

Type	Primers	Sequence (5ʹ–3ʹ)	Source
External primer	F3	GAAAGCATGGGTAGAGGTG	The previous study (Khammanee et al., 2021)
	B3	TTGTTCAACGGAATCTAAT	
Internal primers	FIP* (F1c–F2)	*FITC*-ACCATTAGTTCCACCAATGAC-CCCCTAGATCATCAGCTATGc**A**	Modified from the previous study (Khammanee et al., 2021)
	BIP* (B1c–B2)	*Biotin*-CCATATGCCTTATTAGAAG-CTCCAACAACATATATTTGATTAAGG	
Loop primer	LF	TTTATTATCAAAAGCAACg**T**	This study

Note: Nucleotides in frames are mismatches added to specifically distinguish the C580Y mutation. Nucleotides in bold are specific primers with the mutation. F3, forward external primer; B3, backward external primer; FIP*, 5ʹ end of FIP primer labeled with fluorescein isothiocyanate; BIP*, 5ʹ end of BIP primer labeled with biotin; LF, forward loop primer.

### 2.3 Preparation of chromatographic lateral flow assay

**Fig. 1:**
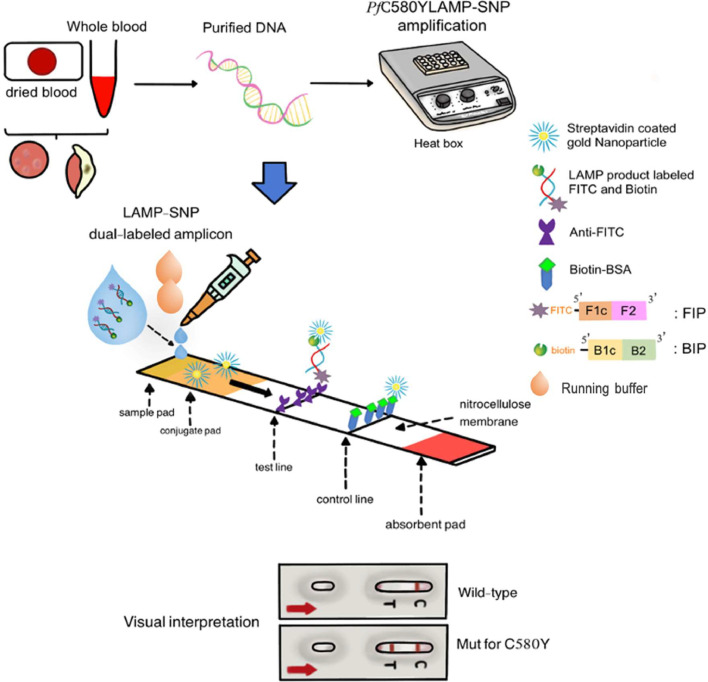
Schematic illustration of the process of the *Pf*C580Y LAMP-SNP-LFA method, the structure of the chromatographic lateral flow assay, and its interpretation.

A lateral flow device was designed to detect the *Pf*C580Y mutation using fluorescent molecules to label the primers labeled with FITC and biotin (Fig. 1). First, the *Plasmodium* DNA sample was amplified using the LAMP-SNP method, with the 5ʹ ends of the FIP and BIP primers labeled with FITC and biotin, respectively (Table 1) to produce a detectable dual-labeled amplicon. Second, the resultant LAMP product was placed into a lateral flow device that contained a nitrocellulose membrane coated with streptavidin-coated nanogold particles, which immobilized the dual-labeled amplicon and allowed it to flow through to the next zone. Third, the detection zone was coated with anti-FITC antibodies on the test line, which recognized the FITC label on the amplicon, forming an antibody-amplicon complex on the test line if the target DNA sequence was present in the sample. This produced a visible colored band on the strip. At the same time, any excess non-target products and streptavidin-conjugated gold nanoparticles were captured with biotinylated bovine serum albumin (BSA) embedded on the control line. The results were visualized within 5 min. Two red lines, one at the test line and one at the control line, indicated a positive result, while one red line at the control line indicated a negative result.

### 2.4 Sensitivity and specificity of *Pf*C580Y LAMP-SNP-LFA method and conventional PCR assays

**Fig. 2:**
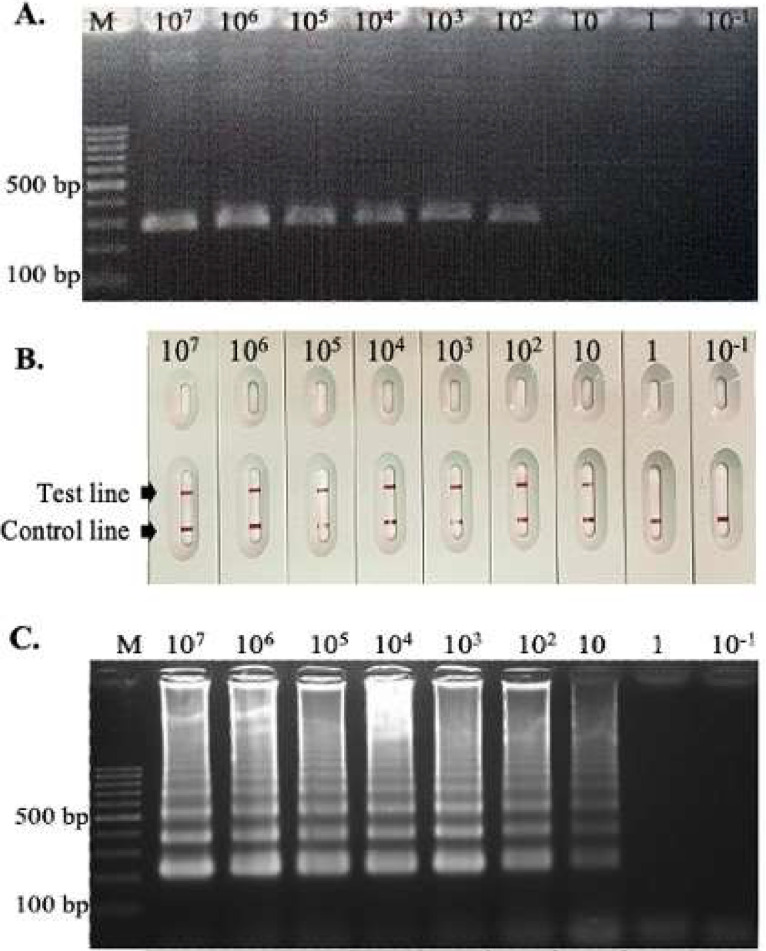
Comparison of the sensitivity of the conventional PCR assay (A) with the LAMP-SNP-LFA method (B) using 10-fold serial dilutions of *PfC580Y* plasmid DNA. (A) Agarose gel electrophoresis was performed on the resultant amplicons from conventional PCR using primers F3 and B3 primers. (B) The results of the *Pf*C580Y LAMP-SNP-LFA method were detected with a LFA strip test. (C) Agarose gel electrophoresis of 5 µL of the *Pf*C580Y LAMP-SNP-LFA product. M: 100 bp DNA Ladder (Thermo Scientific). Lanes 1–9: 1.68 × 10^7^, 1.68 × 10^6^, 1.68 × 10^5^, 1.68 × 10^4^, 1.68 × 10^3^, 1.68 × 10^2^, 1.68 × 10^1^, 1.68 × 10^0^, and 1.68 × 10^–1^ copies of DNA template/µL, respectively. Sterile distilled water and plasmids carrying *Pfkelch13* wild-type were used as templates for the negative control in the reactions.

**Fig. 3:**
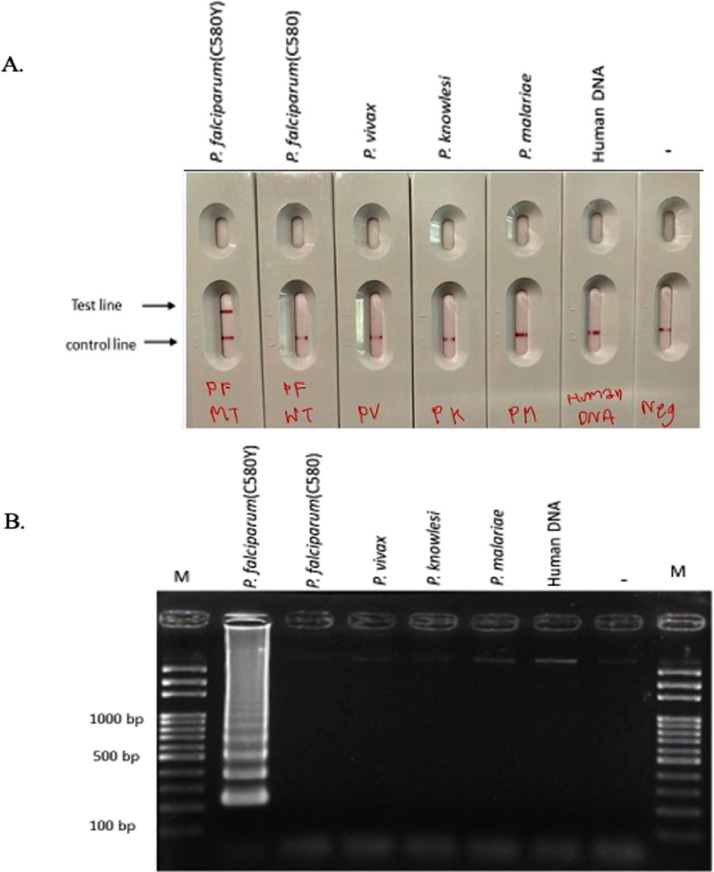
Specificity analysis of our LAMP-SNP-LFA method for *Pfkelch13* C580Y mutation detection. (A) Specificity of the *Pf*C580Y LAMP-SNP-LFA method using a LFA strip test and (B) agarose gel electrophoresis of 5 µl of the *Pf*C580Y LAMP-SNP-LFA product. M: 100 bp DNA ladder Lane 1: *Pfkelch13* mutant-type (positive plasmid control), 2: *Pfkelch13* wild-type (negative plasmid control), 3: *P. vivax* DNA, 4: *P. knowlesi* DNA, 5: *P. malariae* DNA, 6: human DNA, and 7: no DNA template.

The sensitivity of the LAMP-SNP-LFA method was compared with the conventional PCR assay using the F3 and B3 primers and evaluated using a plasmid carrying the C580Y mutation. The template was prepared by making 10-fold serial dilutions (1.68 × 10^7^–1.68 × 10^–1^ copies/µL of plasmid DNA) to ascertain the limit of detection. PCR amplification was performed according to the protocol described in the previous study. Briefly, the PCR assay was performed in final volume of 25 µL that contained 1 × PCR buffer (Invitrogen Life Technologies, CA), 2 mM of MgCl_2_, 0.25 mM of each dNTP, 1 U of *Taq* DNA polymerase, and 1 µL of plasmid DNA template. The PCR cycling conditions were as follows: denaturation at 94 °C for 5 min; 35 cycles of 94 °C for 1 min, 56 °C for 1 min, and 72 °C for 2 min; and final extension at 72 °C for 5 min. PCR was performed in a T100 Thermal Cycler (Bio-Rad, USA) (Khammanee et al., 2021). The PCR products were separated by electrophoresis on a 2% agarose gel and observed under UV transillumination. To evaluate the specificity of the *Pf*C580Y LAMP-SNP-LFA assay, the following DNA templates were used: *Pfkelch13* C580Y mutant-type, *Pfkelch13* wild-type, *P. vivax, P. malariae*, and human DNA. The sensitivity and specificity of *Pf*C580Y LAMP-SNP amplification were analyzed individually using the LFA test strip and confirmed using 2% agarose gel electrophoresis (Fig. 2 and 3).

### 2.5 Practical application of LAMP-SNP-LFA method to detect *Pf*C580Y mutation from genomic DNA isolated from clinical samples

The *Pf*C580Y LAMP-SNP-LFA method was utilized to identify the *Pf*C580Y mutation in 100 DNA samples that were randomly selected in a blinded manner from two prior studies (Imwong et al., 2015; Khammanee et al., 2019). The sample set consisted of 19 wild-type *Pfkelch13* samples, 72 *Pfkelch13* samples with the C580Y mutation, 8 *P vivax* samples, and 1 P *malariae* samples. The *Plasmodium* species of each sample had already been confirmed, and nested PCR followed by DNA sequencing had been performed to determine whether the *Pfkelch13* C580Y mutation was present.

The *Pf*C580Y LAMP-SNP-LFA method was performed in a total reaction volume of 25 µL consisting of 2.5 µL of 10 × buffer (New England Biolabs), 2 mM MgSO_4_, 0.4 M betaine (Sigma-Aldrich), 1 mM dNTP mix, 0.2 µM each of F3 and B3 primers, 1.6 µM each of FIP* and BIP* primers, 0.2 µM of LF primer, 8 U of *Bst* DNA polymerase (New England Biolabs), and 2 µL of template DNA. The LAMP-SNP-LFA method involved incubation for 45 min at 56 °C in a heat block, followed by incubation at 82 °C for 2 min to terminate the reaction. Two different methods were used to analyze the results. The first method utilized 2% agarose gel electrophoresis, followed by staining with 0.5 µg/mL ethidium bromide and observing the amplicons under UV transillumination. The second method involved LFA test strips, where 5 µL of biotin-dsDNA-FITC samples was added to the sample pad, followed by 100 µL of running buffer (10 mM PBS [pH 7.4] with 1% Tween 20). The results were visualized within 5 min (positive, two red lines: test line and control line; negative, one red line: control line).

### 2.6 Statistical analysis

The sensitivity, specificity, positive predictive value (PPV), and negative predictive value (NPV) of the LAMP-SNP-LFA method for *Pf*C580Y mutation detection were compared with the results obtained from DNA sequencing, which served as the reference method. Data analysis was performed using MedCalc statistical software (https://www.medcalc.org/calc/diagnostic_test.php) with 95% confidence intervals (CIs). The assay sensitivity (%) was calculated as follows: [true positives (TP) / TP + false negatives (FN)] × 100. Similarly, specificity (%) was calculated as follows: [true negatives (TN) / TN + false positives (FP)] × 100. The PPV was computed as (TP / TP + FP) × 100 and the NPV as (TN / (TN + FN) × 100.

## 3 Results

### 3.1 Analytical sensitivity and specificity

The conventional PCR assay exhibited a detection limit of 100 plasmid copies/µL (Fig. 2A). In contrast, the *Pf*C580Y LAMP-SNP-LFA method demonstrated a 10-fold higher sensitivity compared with the conventional PCR assay, showing a distinctly visible red color on both the test and control lines at a plasmid concentration of 10 copies/µL (Fig. 2B). The LFA result was confirmed via agarose gel electrophoresis of a 5 µL aliquot of the *Pf*C580Y LAMP-SNP-LFA product (Fig. 2C). Thus, the *Pf*C580Y LAMP-SNP-LFA technique showed a tenfold increase in efficiency compared with conventional PCR.

The specificity of the *Pf*C580Y LAMP-SNP-LFA method was assessed by testing it with plasmid templates carrying *Pfkelch13* C580Y mutant-type and *Pfkelch13* wild-type, as well as DNA templates of *P. vivax, P. malariae*, and human DNA. All samples tested negative (one red line: control line) except for the *Pfkelch*13 C580Y mutant, as indicated by a visible signal on both the test and control lines. This observation was consistent with the electrophoresis results (Fig. 3A and B).

### 3.2 Application of *Pf*C580Y LAMP-SNP-LFA method to clinical samples

We validated the diagnostic performance of the LAMP-SNP-LFA method for detecting the *Pfkelch13* C580Y mutation using DNA samples of *Plasmodium* species taken from samples of 100 malaria patients. Of them, 62 DNA samples were obtained from the whole blood of *P. falciparum*-infected patients (*PfC580Y* mutation, 50, and *Pf* wild-type, 12) (Imwong et al., 2015). The remaining 38 samples were obtained from dried blood spots, including 29 *P falciparum* samples (*Pf*C580Y mutation, 22, and *Pf* wild-type, 7), 8 *P vivax* samples, and 1 *P malariae* sample (Khammanee et al., 2019). The average DNA concentration in whole blood samples was 27.1 ng/µL (range:8.4–96.8 ng/µL), while the average parasite density was 17,018/µL (range: 5000–125,000 parasites/µL), with six samples lacking malaria parasite density data. The parasite density in the dried blood spot samples was 6555/µL (range: 67–84,000 parasites/µL), with 17 missing density data (*P. falciparum*, 8; *P. vivax*, 8; and *P. malariae*, 1). The blood spot samples (50–80 µL) used in this study were collected from 2014 to 2019. The DNA quality and stability of these samples were negatively affected due to inadequate protection against humidity resulting from the infrequent replacement of silica beads and the potential degradation caused by higher temperatures and increased humidity, including freeze-thaw cycles at room temperature (Hwang et al., 2012). To address this issue and increase the DNA concentration of the dried blood spot samples, a speed vacuum centrifuge was utilized to reduce the volume of the samples and dry them (Imwong et al., 2014). Thereafter, they were resuspended in 20 µL of PCR-grade water. This process resulted in an average increase in DNA concentration from 4.71 ng/µL before speed vacuum centrifugation to approximately 22.1 ng/µL. This study utilized LAMP-SNP-LFA technology, which was specifically designed for clinical applications, to detect the C580Y mutation and interpret the findings based on colorimetric changes on the LFA strip, thereby reducing the potential for inaccuracies resulting from visual interpretation using dyes. Additionally, gel electrophoresis was employed to confirm successful amplification through the presence of a specific ladder-like band pattern. Table 2 compares the results of the *Pf*C580Y LAMP-SNP-LFA method with the DNA sequencing results. Notably, the *Pf*C580Y LAMP-SNP-LFA method detected the C580Y mutations at a parasite density of 67/µL, which was the lowest parasite density observed among the collected samples.

**Table 2:** Evaluating the detection of the *Pf*C580Y mutation using the LAMP-SNP-LFA method in 100 DNA samples from clinical specimens.

*Plasmodium* species	DNA sequencing^a^	No. of samples	Parasite density range (parasite/µL)	Interpretation of PfC580Y LAMP-SNP-LFA method
				(+, positive; −, negative)
*P. falciparum*	C580Y	4	67–800	+
	C580Y	7	1733–4230	+
	C580Y	16	5000–8000	+
	C580Y	15	10,000–12,078	+
	C580Y	9	15,000–15,428	+
	C580Y	11	20,000–40,000	+
	C580Y	4	50,000–48,000	+
	C580Y	1	125,000	+
	C580Y	5	ND	+
	wild-type	3	840–2069	−
	wild-type	3	5000–5137	−
	wild-type	5	10,000–40,000	−
	wild-type	8	ND	−
*P. vivax*	-b	8	ND	−
*P. malariae*	-b	1	ND	−

a DNA sequencing data were obtained from two previous studies (Imwong et al., 2015 and Khammanee et al., 2019).

b The protocol employed for the identification of *Plasmodium* species in this study was based on the methodology described by Snounou and colleagues (Snounou et al., 1993). ND; no data.

### 3.3 Comparison of sensitivity and specificity of the *Pf*C580Y LAMP-SNP-LFA method with the standard DNA sequencing method

A total of 91 samples of *P. falciparum* were included in this analysis (*Pf*C580Y mutation, 72, and *Pf* wild-type, 19). The sensitivity of the *Pf*C580Y LAMP-SNP-LFA test was 100% (95% CI: 94.94–100.0) and was calculated as follows: [TP/(TP + FN)] × 100 = 72/(72 + 0) × 100 = 100% (72/72 positives). The specificity was also 100% (95% CI: 83.16–100.0) and was calculated as follows: (TN) / (FP + TN) × 100 = 19/(0 + 19) × 100 = 100% (19/19 negatives). The PPV was 100%: [TP/(TP + FP)] × 100 = 72/(72 + 0) × 100 = 100%, and the NPV was 100%: [TN / (TN + FN)] × 100 = 19/(19 + 0) × 100 = 100%. The analysis is presented in Supplementary Table 1. Additionally, nine non-*P. falciparum* samples tested negative with our *Pf*C580Y LAMP-SNP-LFA method.

Our results demonstrate that the *Pf*C580Y LAMP-SNP-LFA technique is capable of accurately detecting the *Pfkelch13* C580Y mutation in clinical samples. This allows for precise differentiation between strains with the mutation associated with ART resistance and the wild-type.

## 4 Discussion

Previous studies have developed a LAMP-SNP technique for detecting the *Pfkelch13* C580Y mutation (Imai et al., 2018; Khammanee et al., 2021; Mohon et al., 2018). However, some of these studies had limitations, such as the need for expensive laboratory equipment like the MinION device, to accurately interpret results. Some studies. used fluorescent or colorimetric indicators and interpreted the results with the naked eye. Nevertheless, the use of these methods which involve preparing working stock solutions from stock solutions, may lead to contamination, resulting in potential false–positive or false–negative results.

Additionally, insufficient amplicon formation in LAMP may lower the reaction's pH, affecting color changes and leading to misinterpretation of results. The effectiveness of the LAMP reaction with fluorescent or colorimetric indicators can also be influenced by the concentrations of *Bst* DNA polymerase, MgSO_4_, internal and external primers, and betaine. Therefore, the indicator used for interpreting LAMP results may not be sufficiently specific for an accurate interpretation of the reaction (Aoki et al., 2021; Notomi et al., 2000; Sun et al., 2017; Wang et al., 2015).

In this study, the LFA method was employed for result interpretation, based on the principles of chromatography and specific labeling of antibodies to elicit a reaction on a nitrocellulose membrane, allowing for rapid flow and permeation of the solution. In the *Pf*C580Y LAMP-SNP-LFA method, the 5ʹ end of the FIP primer is labeled with FITC, and the FIP primer also includes a mismatched nucleotide ensuring specific capture of the C580Y mutation. The 5ʹ end of the BIP primer is labeled with biotin, and a loop primer with a mismatched nucleotide at the 3ʹ end is added to enhance C580Y capture. The use of this straightforward and rapid color strip reading approach allowed the visualization of results within 5 min, minimizing the risk of false positives that can arise due to indicator color contamination or variability in pH buffer values.

The *Pf*C580Y LAMP-SNP amplicon product is labeled with two colors to form an FITC-dsDNA-biotin complex, which is then applied onto the sample pad with a running buffer. The FITC-dsDNA-biotin amplicon is captured by the anti-FITC antibody on the test line of the nitrocellulose membrane, while the streptavidin-bound gold nanoparticles from the running buffer specifically capture the biotin of the FITC-dsDNA-biotin amplicon, forming a complex that produces a red color on the test line. The remaining buffer flows to the control line, where the streptavidin is captured by biotin-labeled BSA, producing a red color. If no FITC-dsDNA-biotin complex is formed in the reaction, only the control line turn red, indicating the absence of the C580Y mutation. In our study, we compared the sensitivity of the LAMP-SNP-LFA method to conventional PCR with a single pair of external primers (F3/B3). The LAMP-SNP-LFA method exhibited a 10-fold greater efficiency compared with conventional PCR (16.8 ng/µL vs. 168 ng/µL). This improved sensitivity can be attributed to several factors. First, our study employed the LAMP reaction using a set of five primers that specifically targeted multiple regions of the DNA sequence of interest. This approach enhanced the likelihood of successful amplification, even in the presence of low target concentrations or inhibitory substances. Moreover, the LAMP method achieved a high degree of amplification efficiency due to the presence of loop structures within the amplification products, which acted as templates for primer binding and subsequent amplification, resulting in the rapid accumulation of target DNA molecules. Additionally, the isothermal conditions of the LAMP reaction, typically maintained at a constant temperature (approximately 56 °C), eliminated the need for thermal cycling, reducing the risk of sample loss or degradation during temperature fluctuations.

This method successfully detected the *Pfkelch13* C580Y mutation at malaria parasite densities as low as 67/µL. Notably, this was the lowest parasite density observed among the analyzed samples. Furthermore, our LAMP-SNP-LFA method is comparable to the widely used microscopic method, which can detect parasite densities of 50–100/µL (Moody, 2002). Thus, the parasite density detection in this study was comparable to that of microscopy, which may be a limitation of our study.

However, we demonstrated the reliability of our method in accurately identifying the *Pfkelch13* C580Y mutation, responsible for ART resistance, within a short timeframe of approximately 110 min, including DNA extraction, LAMP-SNP amplification, and LFA test strip analysis (Supplementary Figure 1). Therefore, our method not only reliably and rapidly identified this critical mutation but also showed the potential for utilization in near-to-patient settings, paving the way for more effective malaria surveillance strategies.

The turnaround time of our LAMP-SNP-LFA method is approximately eight times shorter than SNP typing using the PCR/DNA sequencing method, which also requires expensive PCR reagents and sophisticated equipment. Moreover, our method is estimated to cost approximately USD $14 per reaction, which is less than the cost of the PCR/DNA sequencing-based SNP genotyping method. Additionally, the LAMP-SNP-LFA method offers enhanced visualization compared to other methods with the colorimetric method, turbidity, and separation by agarose gel electrophoresis (Park, 2022). The *Pf*C580Y LAMP-SNP-LFA method is advantageous for point-of-care diagnostics in remote areas with a high prevalence of malaria because it only requires basic equipment and a simplified DNA extraction process.

## 5 Conclusions

In summary, the combination of the *PfC580Y* LAMP-SNP method with the LFA technique provides a rapid and accurate tool for detecting the *Pfkelch13* mutation associated with ART resistance. The application of this method can support malaria elimination policies and help in monitoring the spread of drug-resistant infections. Furthermore, this method overcomes the limitations of previous techniques that require expensive laboratory equipment, have a potential for false-positive or false-negative results, and may lead to misinterpretation of results. Hence, this innovative approach provides a reliable and cost-effective method of detecting malaria mutations and combating the global burden of this disease.

## Financial support

This study was supported by Thailand Science Research and Innovation (TSRI) and National research council of Thailand, RTA6280006 and part of the Mahidol-University Oxford Tropical Medicine Research Programme funded by the Wellcome Trust of the UK (core grant 106698/B/14/Z) and Wellcome OA statement. This research was funded in whole, or in part, by the Wellcome Trust [200211]. For the purpose of Open Access, the author has applied a CC BY public copyright licence to any Author Accepted Manuscript version arising from this submission. The funding sources did not participate in data analysis or the final decision to publish the manuscript.

## Ethical standards

The study obtained ethical approval from the Office of Human Research Ethics Committee, Health Sciences, Prince of Songkla University (approval no: HSc-HREC63–6–1–1). Approval was also obtained from the Ethical Review Committees of the Faculty of Tropical Medicine, Mahidol University (approval no: MUTM 2012–045–05 and MUTM 2023–015–01). All study subjects provided informed consent prior to participation.

## Author statement

Wannida Sanmoung: Methodology, Writing- Original draft preparation. Supinya Thanapongpichat: Conceptualization, Writing- Original draft preparation, Writing- Reviewing and Editing. Nongyao Sawangjaroen: Resources, Conceptualization, Suwannee Jitueakul: Resources, Methodology. Hansuk Buncherd: Writing- Original draft preparation. Aung Win Tun: Writing- Original draft preparation, Writing- Reviewing and Editing. Mallika Imwong: Resources, Writing- Original draft preparation, Writing- Reviewing and Editing. All authors read and approved the final manuscript.

## Declaration of Competing Interest

The authors declare that they have no known competing financial interests or personal relationships that could have appeared to influence the work reported in this paper.

## Data Availability

No data was used for the research described in the article.

## Appendix Supplementary materials

Image, application 1

Image, application 2
