# Supplementary material for: Application of loop-mediated isothermal amplification combined with lateral flow assay visualization of Plasmodium falciparum kelch 13 C580Y mutation for artemisinin resistance detection in clinical samples
Source: Acta Trop. 2023 Oct;246:106998. doi: 10.1016/j.actatropica.2023.106998 (PMC10465885; doi:10.1016/j.actatropica.2023.106998)

**Supplementary Figure 1.** Evaluation of the *Pf*C580Y LAMP-SNP-LFA method comparing its results with their individual DNA sequencing data. The success of the LAMP-SNP-LFA amplification was confirmed by the presence of a specific ladder-like pattern following agarose gel electrophoresis. Mut., *Pf*C580Y mutation; WT, *Pfkelch13* wild-type.


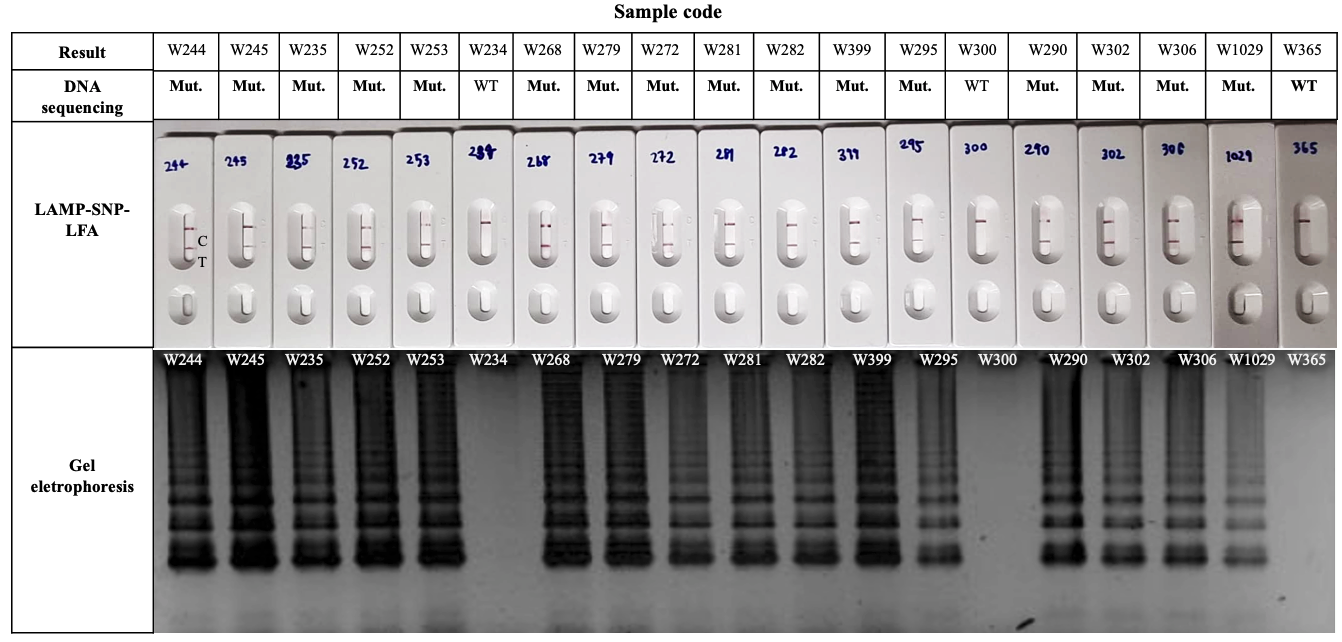


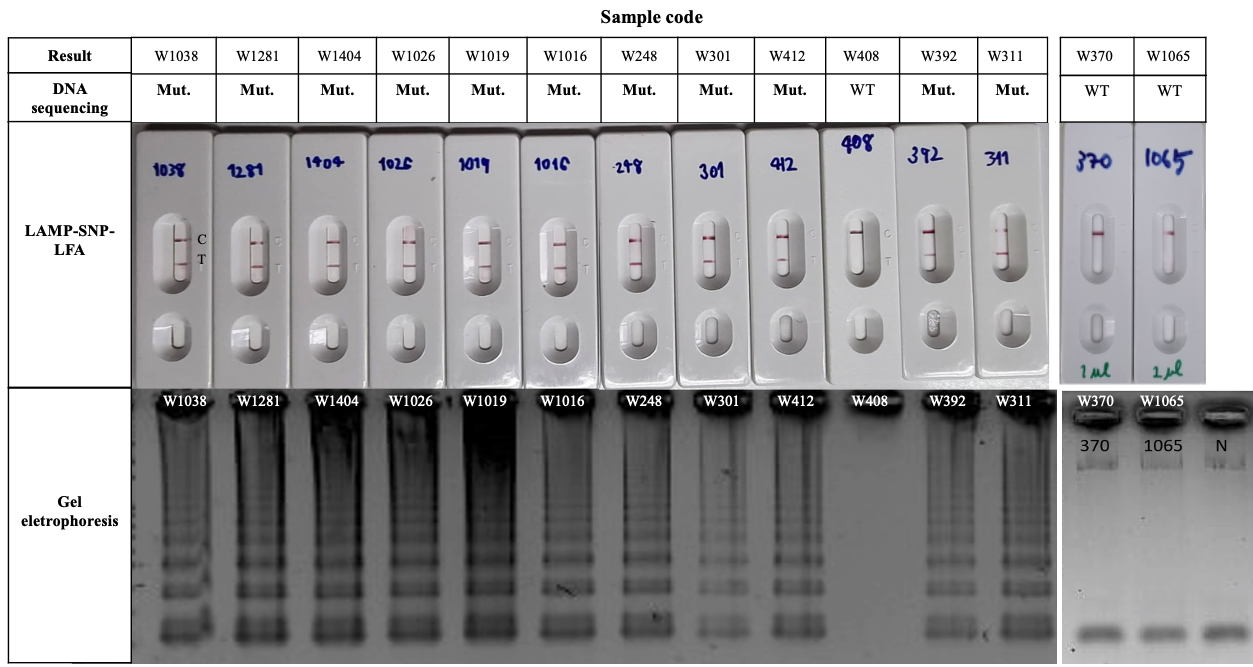


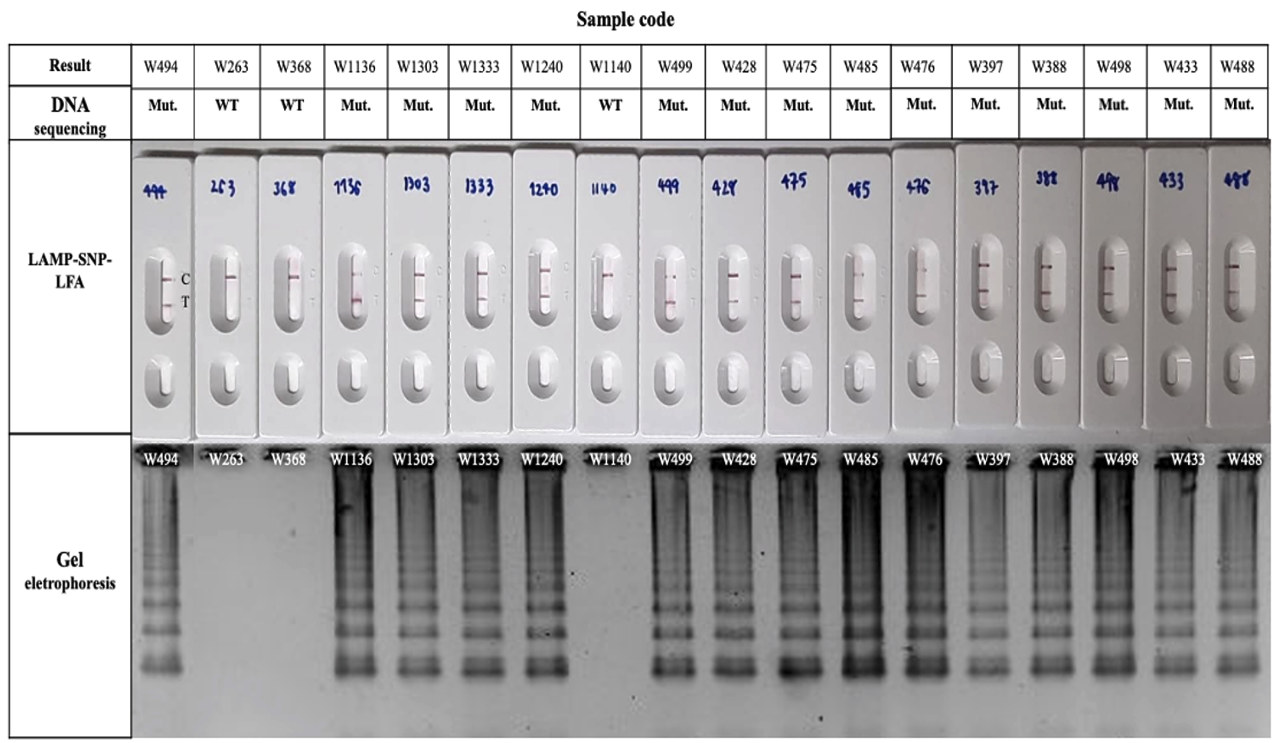


**
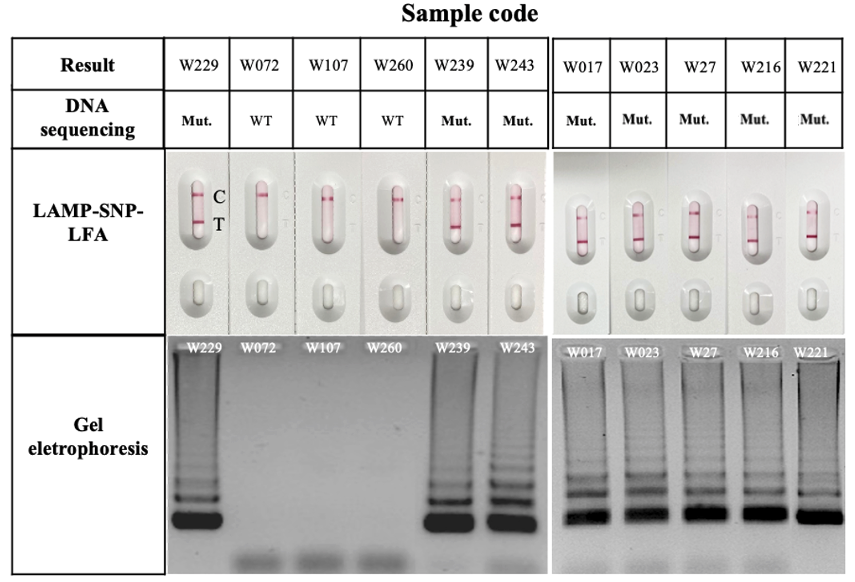
**


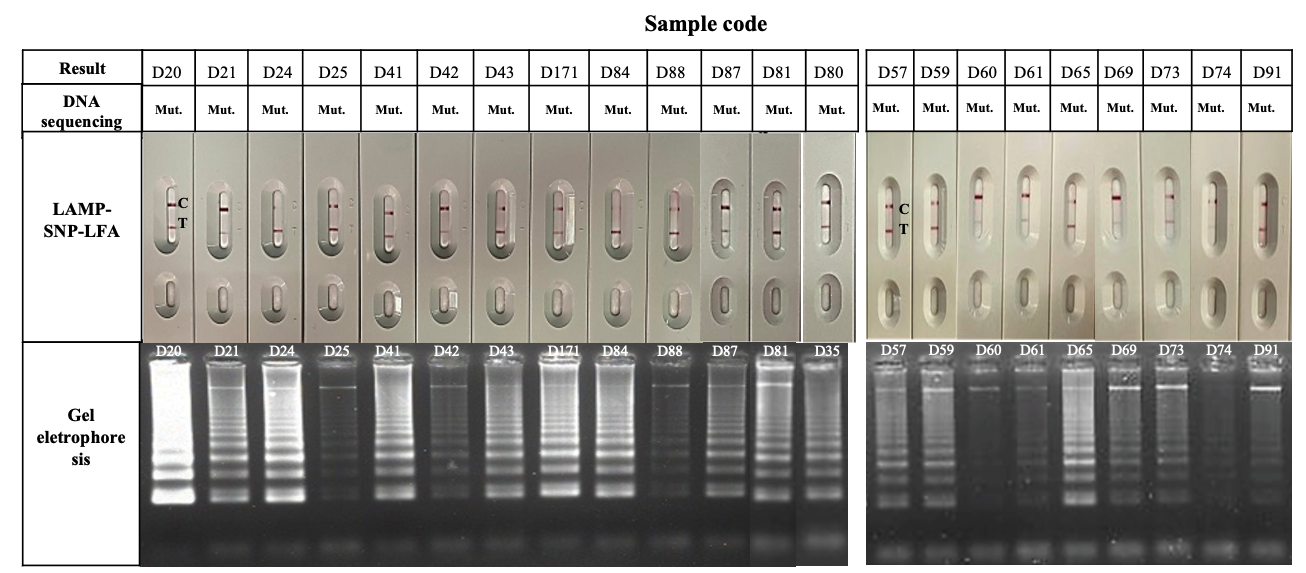


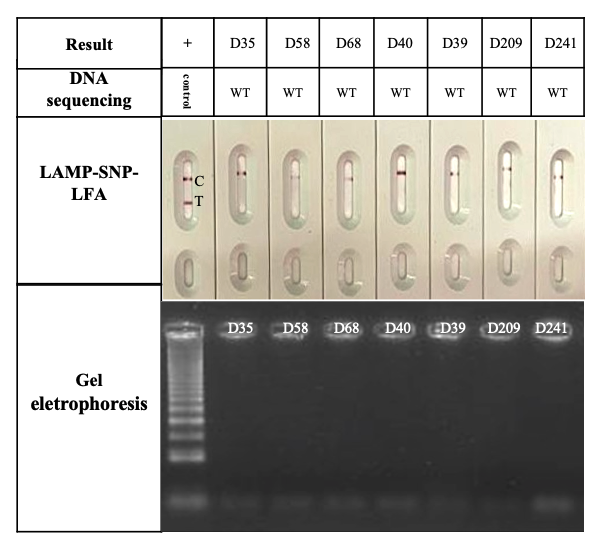

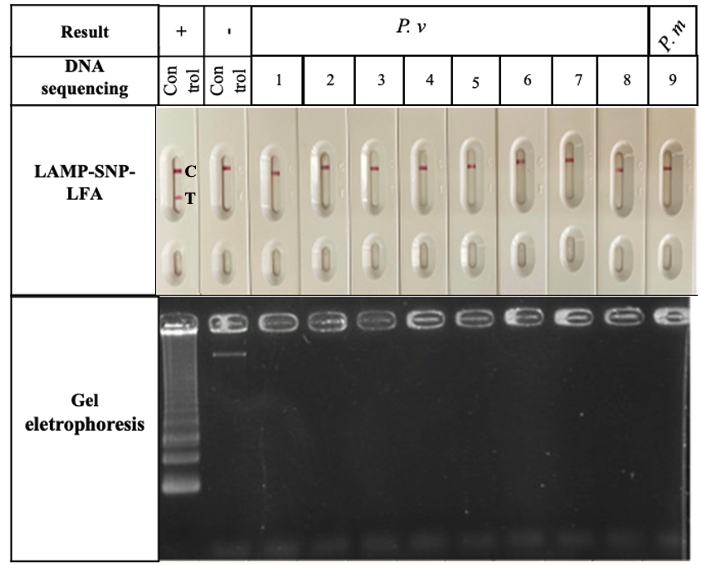

Supplement: Supplementary file 1 [file mmc1.docx]
