# Supplementary material for: Application of loop-mediated isothermal amplification combined with lateral flow assay visualization of Plasmodium falciparum kelch 13 C580Y mutation for artemisinin resistance detection in clinical samples
Source: Acta Trop. 2023 Oct;246:106998. doi: 10.1016/j.actatropica.2023.106998 (PMC10465885; doi:10.1016/j.actatropica.2023.106998)
Supplement: Supplementary file 2 [file mmc2.docx]

Supplement Table 1. The accuracy of *Pf*C580Y LAMP-SNP-LFA method using DNA sequencing method as the Reference Standard

| *Pf*C580Y LAMP-SNP-LFA method  (This study) | DNA sequencing method | | Total | Sensitivity (%) | Specifcity (%) | PPV (%) | NPV (%) | Accuracy |
| --- | --- | --- | --- | --- | --- | --- | --- | --- |
|  | (Reference Standard) | |  |  |  |  |  |  |
|  | Mutation (C580Y) | Wild Type |  | 95% Confidence Interval | | | | |
| Positive | 72 | 0 | 72 | 100 | 100 | 100 | 100 | 100 |
| Negative | 0 | 19 | 19 |  |  |  |  |  |
| Total | 72 | 19 | 91 | (95.01 - 100.0) | (82.35 - 100.0) | (0.00) | (0.00) | (96.03 - 100.00) |
